# Stanniocalcin2 acts as an anorectic factor through activation of STAT3 pathway

**DOI:** 10.18632/oncotarget.19412

**Published:** 2017-07-20

**Authors:** Yang Jiao, Jiejie Zhao, Guojun Shi, Xing Liu, Xuelian Xiong, Xiaoying Li, Huijie Zhang, Qinyun Ma, Yan Lu

**Affiliations:** ^1^ Department of Endocrine and Metabolic Diseases, Shanghai Clinical Center for Endocrine and Metabolic Diseases, Shanghai Institute of Endocrinology and Metabolism, Ruijin Hospital, Shanghai Jiao Tong University School of Medicine, Shanghai 200025, China; ^2^ Department of Endocrinology, Fudan Institute for Metabolic Diseases, Zhongshan Hospital, Fudan University, Shanghai 200032, China; ^3^ Department of Endocrinology and Metabolism, Nanfang Hospital, Southern Medical University, Guangzhou 510515, China

**Keywords:** appetite, body weight, stanniocalcin 2, STAT3

## Abstract

The regulation of food intake and body weight has been hotly investigated. In the present study, we show that stanniocalcin2 (STC2), a cytokine ubiquitously expressed and especially upregulated in many types of human cancers, has a regulatory role in food intake and weight loss. Systemic treatment of C57BL/6 mice with recombinant STC2 protein resulted in decreased food intake and body weight, whereas energy expenditure was not affected. Similarly, STC2 treatment also induced anorexia in hyperphagic leptin-deficient mice, leading to a significant reduction in body weight and improvement of blood glucose levels. Furthermore, intracerebroventricular administration of STC2 to mice led to an acute decrease in food intake, which was mediated, at least in part, by activation of STAT3 pathway. Taken together, our results revealed the importance of STC2 in the regulation of feeding behavior as well as body weight.

## INTRODUCTION

The maintenance of body weight is a complicated process which is coordinated by energy intake and energy expenditure. While energy expenditure exceeds energy intake, a negative energy balance induced by anorexia or cachexia leads to wasting, debility and even death.

Stanniocalcin (STC) was initially discovered as an endocrine factor which was secreted by endocrine glands and specifically regulated serum calcium/phosphate homeostasis in the bony fish [1–3]. It was not until 1995 that the mammalian form of STC was discoverd by the successful cloning of mouse and human STC cDNA [4–5]. Two years later, when a new member of STC family, the STC-related protein was identified, the previously reported fish and mammalian STCs were renamed STC1, and the additional member of STC family was named STC2 [6].The mammalian STC2 is expressed virtually in all tissues and regulates various biological processes, such as ion transportation, cell proliferation, reproduction and stress response [7–9]. Besides, recent studies have highlighted its role in tumorigenesis. It is reported that STC2 is highly expressed in many human cancer tissues compared with the relevant normal tissues [10–13], which is possibly responsible for poor prognostic outcome [10–13].

It has been well-established that anorexia and weight loss, the main characteristics of wasting syndrome in advanced cancers, could be mediated by cytokines [14–17]. Since STC2 is aberrantly hypersecreted in many human cancers and associated with poor prognosis, we hypothesize that STC2 might be a novel anorexic factor in patients with advanced cancers.

## RESULTS

### Systemic STC2 treatment reduces body weight in C57BL/6 mice by inhibiting appetite

**Figure 1:**
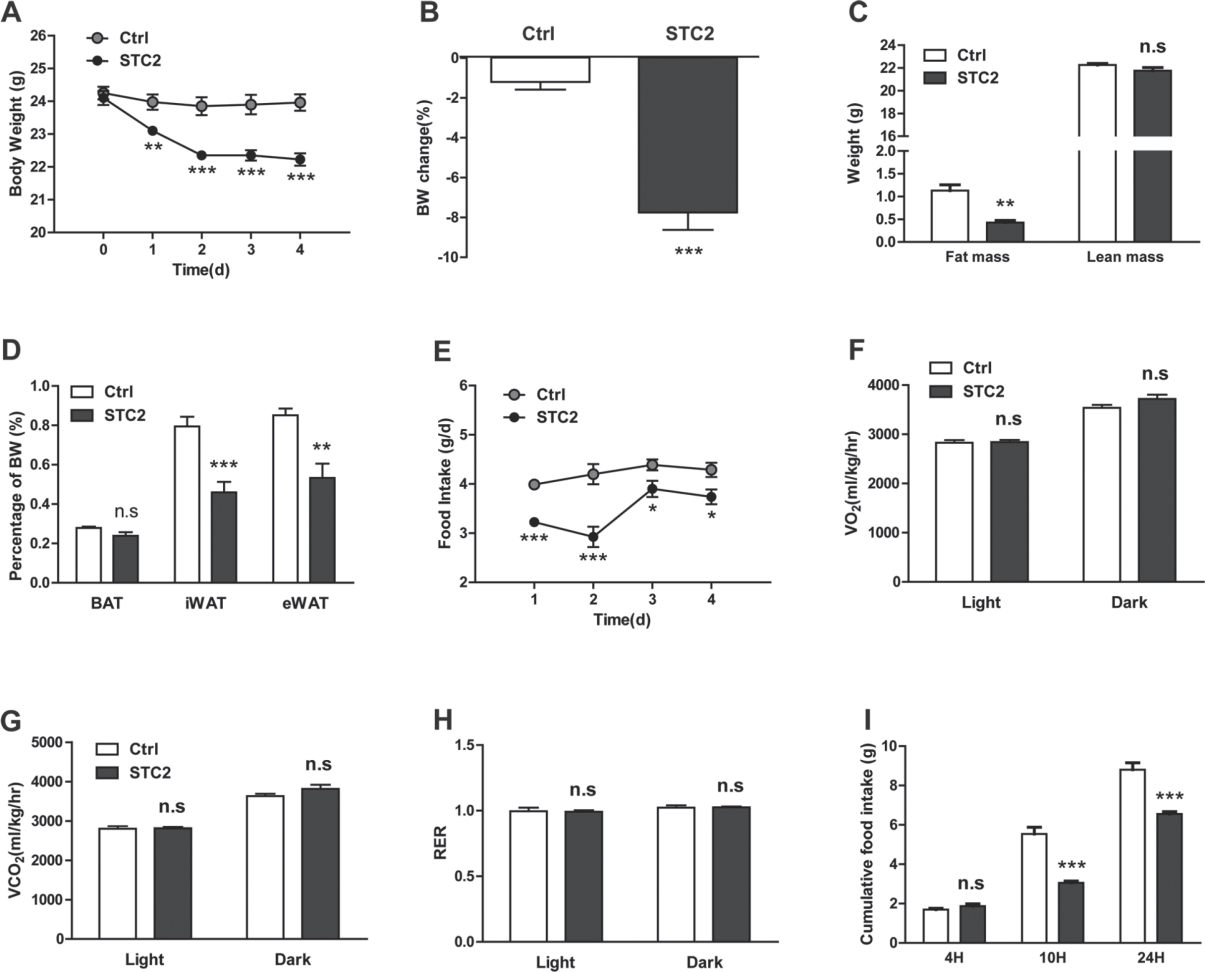
Systemic STC2 treatment reduces appetite and promotes weight loss in C57BL/6 mice (**A**) Body weight monitoring of C57BL/6 mice receiving chronic i.p. injection of STC2 recombinant protein (1.0 mg/kg) or vehicle control (PBS) for 5 successive days (*n* = 8). (**B**) Mice treated with STC2 lost more weight compared to control mice by the end of the experiment (*n* = 8). (**C**) DEXA scanning was performed in two groups of mice (*n* = 4). (**D**) The weight of brown adipose tissue, inguinal and epididymal fat depots were measured in two groups of mice (*n* = 8). (**E**) Daily food intake of mice injected with STC2 or PBS (*n* = 8). (**F–H**) Energy expenditure, determined by O_2_ consumption (F), CO_2_ production (G) and RER (H). (**I**) Cumulative food intake of C57BL/6 mice responding to a single i.p. injection of STC2 recombinant protein (1.0 mg/kg) or PBS after 24 hr of fasting. Data are expressed as means ± SEM. *P < 0.05; **P < 0.01; ***p < 0.001.

To investigate the role of STC2 in food intake, C57BL/6 mice were injected intraperitoneally with recombinant STC2 protein (1.0 mg/kg) twice daily for 5 successive days. Interestingly, mice receiving STC2 administration displayed a progressive weight loss since the first day of injection (Figure 1A). At the end of the experiment (4 days), the body weight was reduced by 7.8%, while in mice receiving PBS the body weight maintained relatively stable (Figure 1B). To characterize the nature of weight loss induced by STC2, we assessed the fat and lean mass of mice by dual-energy X-ray absorptiometry (DEXA) scanning. The result revealed that STC2 primarily induced loss of fat mass rather than lean mass (Figure 1C). A more specific analysis of change in fat mass demonstrated that STC2 treated mice had about 42% and 37% reduction in inguinal and epididymal fat depots, respectively (Figure 1D). However, there was no significant difference in the weight of the inter scapular brown adipose tissue (BAT) depots between groups (Figure 1D). Daily food intake was remarkably reduced (Figure 1E), while energy expenditure remained unaffected, as shown by O2 consumption, CO2 production and respiratory exchange ratios (RER) (Figure 1F–1H).

To further examine the acute effect of STC2, C57BL/6 mice were treated with a single injection of STC2 or vehicle control after fasting for 24 hr. Although in the first 4 hr after injection, there was no significant differences in food intake between the two groups (Figure 1I). However, at 10 hr and 24 hr after the injection, STC2 treated mice consumed much less food compared to PBS treated mice (Figure 1I), further indicating that STC2 treatment could inhibit food intake in mice.

### Systemic STC2 treatment inhibits appetite and reduces body weight in leptin-deficient (ob/ob) mice

**Figure 2:**
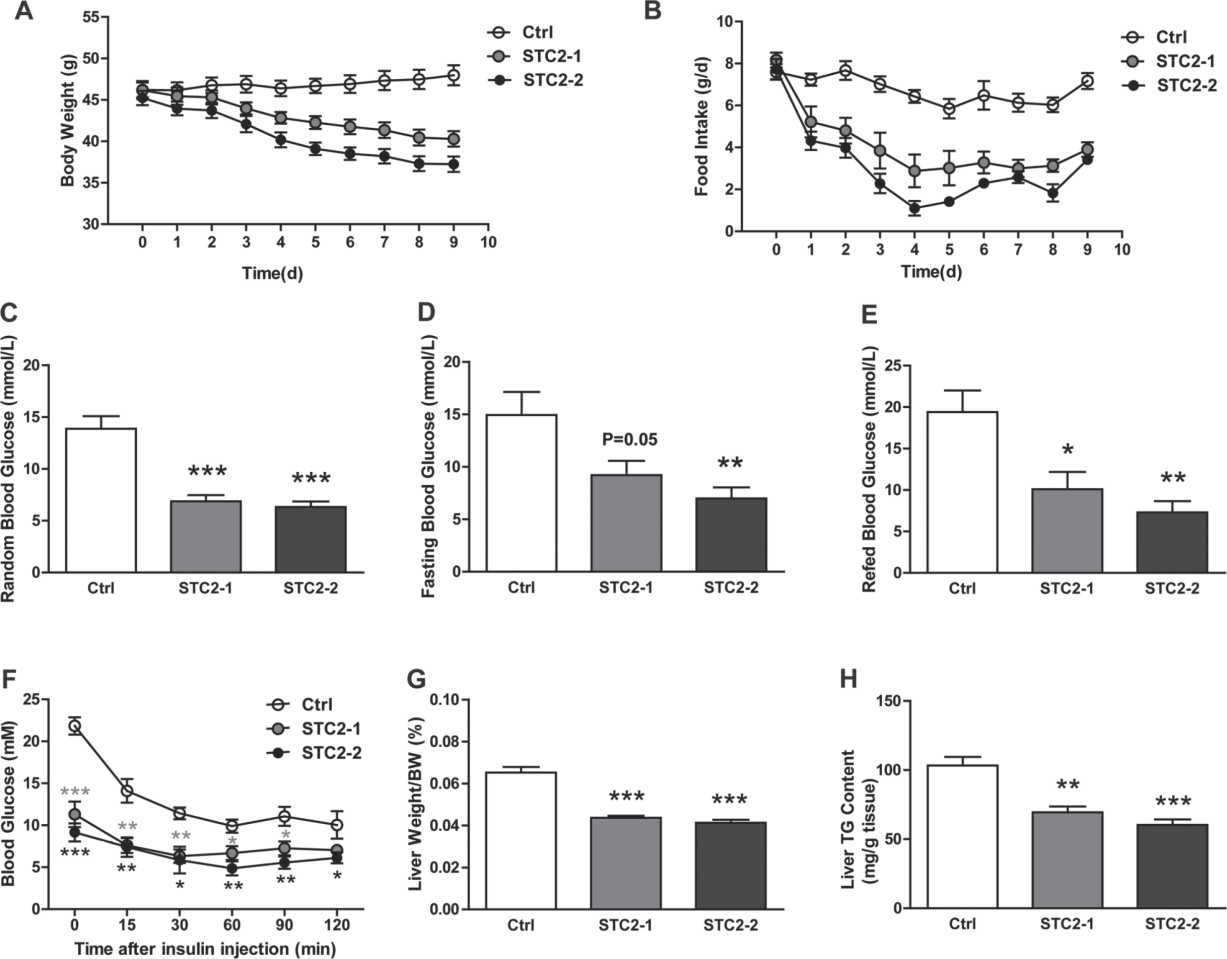
Systemic administration of STC2 reduces appetite and body weight in leptin-defient mice (**A, B**) Body weight and food consumption in ob/ob mice treated with two dose STC2 recombinant protein (STC2-1: 0.5 mg/kg or STC2-2: 1 mg/kg) or vehicle control (PBS) for 10 days (*n* = 6). (**C–E**) Blood glucose levels in random (C), fasting (D) and refed (E) state of ob/ob mice (*n* = 6). (**F**) Insulin tolerance tests in ob/ob mice (*n* = 6). (**G, H**) liver weight/body weight ratios (G) and hepatic triglyceride contents (H) of ob/ob mice (*n* = 6). Data are expressed as means ± SEM. *P < 0.05; **P < 0.01; ***p < 0.001.

To interrogate whether STC2 could have an anorexic role in hyperphagia and obese status, leptin-deficient (*ob/ob*) mice were treated with two different dosages of STC2 (0.5 mg/kg, 1.0 mg/kg). Systemic treatment of *ob/ob* mice with STC2 led to a suppression of daily food intake (Figure 2A) and a dramatic body weight reduction in a dose dependent manner (Figure 2B). Notably, fasting and fed blood glucose levels were reduced in STC2 treated mice (Figure 2C–2E). Insulin tolerance tests showed that blood glucose levels were lower at all-time points, indicating improvement of insulin sensitivity (Figure 2F). In addition, liver weight/body weight ratio and hepatic triglyceride content were also decreased compared to the control group (Figure 2G, 2H). Given that STC2 treatment improved metabolic disorders in ob/ob mice, the mRNA levels of adipogenic genes, hepatic gluconeogenic genes, hepatic triglyceride synthesis related genes were analyzed in *ob/ob* mice. As a result, we found that mRNA levels of adipogenic genes (PPARγ, C/EBPα, C/EBPβ) were downregulated in ob/ob mice treated with higher dosage of STC2 (Supplementary Figure 1A). Besides, PEPCK, the key gluconeogenic gene, was downregulated by STC2 administration (Supplementary Figure 1B). Moreover, expression levels of hepatic lipogenic genes (SREBP-1c, FASN, ACC1, SCD1) were downregulated (Supplementary Figure 1C). Together, these results clearly indicate that STC2 could also lead to anorexia and body weight loss in *ob/ob* mice. In addition, we observed that food intake and weight gain were also inhibited in C57BL/6 mice consuming a high-fat diet (Supplementary Figure 2A, 2B), suggesting a potential role of STC2 in combating obesity and metabolic disease.

### I.c.v. administration of STC2 inhibits acute food intake in C57BL/6 mice

**Figure 3:**
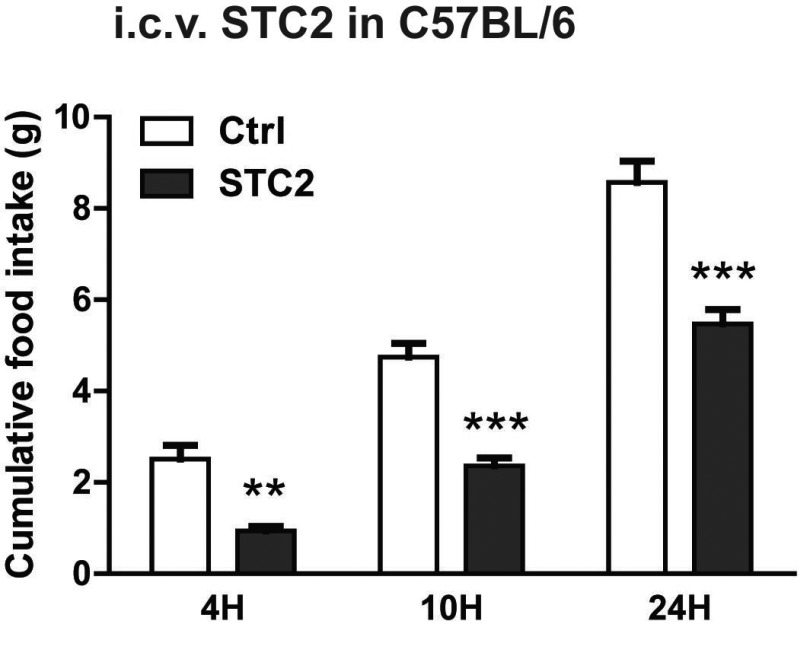
I.c.v. injection of STC2 inhibits acute food intake in C57BL/6 mice. C57BL/6 mice were fasted for 24 hr, then i.c.v. administrated with STC2 recombinant protein (1 μg) or vehicle control (PBS) into the lateral ventricle Cumulative food intake of mice was measured over 4 hr, 10 hr and 24 hr after injection (*n* = 6–8). Data are expressed as means ± SEM. **P < 0.01; ***p < 0.001.

The central hypothalamus is the major center for body weight and energy balance control which senses and integrates various hormonal, neuronal, and nutrient-related signals [18–21]. To further explore whether STC2 mediated anorexia and weight loss was dependent on hypothalamic effect, we administrated recombinant STC2 protein or vehicle control (PBS) into the lateral ventricle of C57BL/6 mice, and monitored their food intake over time. With ad libitum access to food, STC2-treated mice consumed 57.8% less food than control mice over 4 hr after the i.c.v. injection. Moreover, cumulative food intake over 10 and 24 hr was significantly reduced (Figure 3). To be noted, a much earlier anorexic effect was observed and a much lower dose is required when STC2 is injected centrally than peripherally, suggesting STC2 may exert its anorexic effect in the central nervous system(CNS).

### STC2 modulates hypothalamic AGRP, NPY, POMC, CART expression

**Figure 4:**
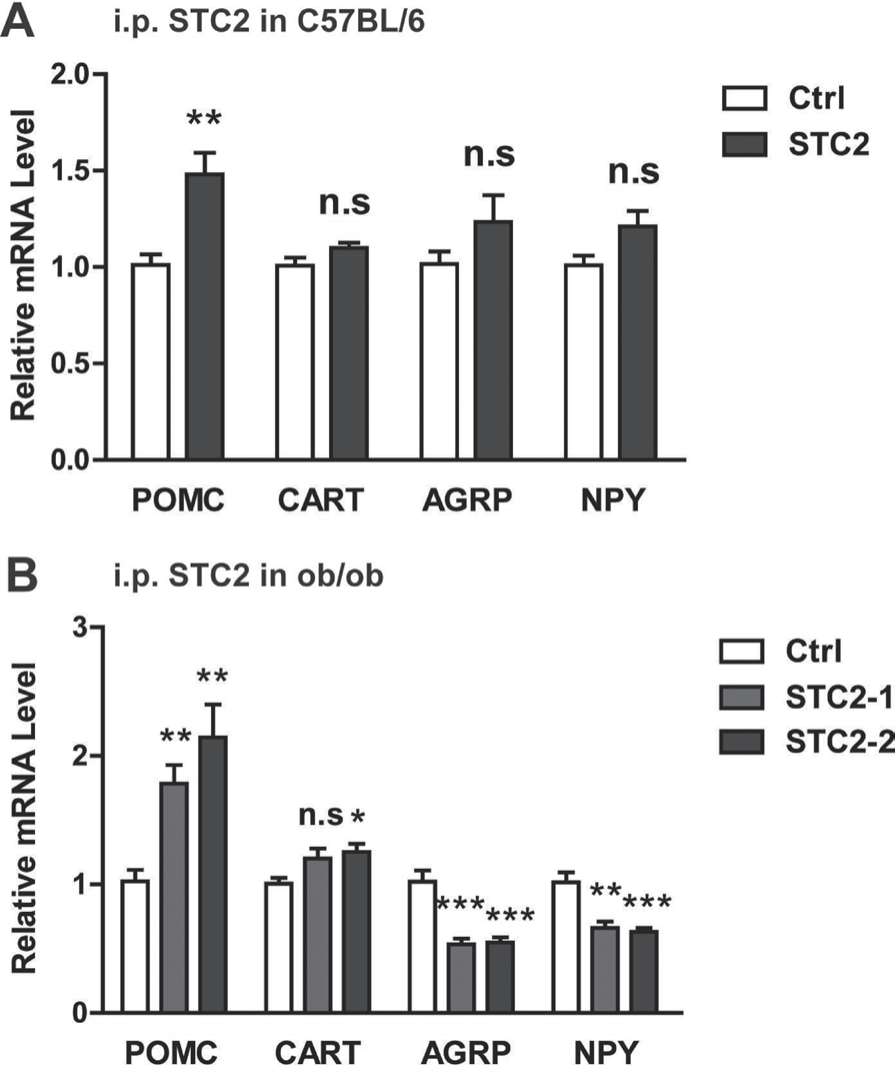
STC2 modulates hypothalamic AGRP, NPY, POMC and CART expression (**A**) Relative mRNA levels of POMC, AgRP, NPY and CART in STC2 i.p. administrated C57BL/6 mice (*n* = 8). (**B**) Relative mRNA levels of POMC, AgRP, NPY and CART in ob/ob mice that received systemic STC2 treatment. Data are expressed as means ± SEM. *P < 0.05; **P < 0.01; ***p < 0.001.

The arcuate nucleus (ARC) in the mediobasal hypothalamus contains two distinct populations of neurons that exert potent effects on food intake and energy expenditure. The orexigenic neurons coexpress agouti-related protein (AgRP) and neuropeptite Y (NPY) to promote caloric intake, while the anorectic neurons coexpress proopriomelanocortin (POMC) and cocaine and amphetamine regulated transcript (CART) neuropeptites to suppress food intake [17]. To determine whether these neuropeptites are involved in the anorexia and weight loss induced by STC2, we examined hypothalamic AgRP, NPY, POMC and CART expression in C57BL/6 and ob/ob mice systemically treated with STC2. As a result, hypothalamic anorexic POMC was elevated, while the expressions of AgRP, NPY and CART were not markedly changed in STC2 i.p. administrated C57BL/6 mice (Figure 4A). Similarly, we also found a significant up-regulation of hypothalamic POMC expression in *ob/ob* mice that received systemic STC2 treatment. Besides, the anorexic CART expression was increased. A dramatic decrease in orexigenic AgRP and NPY expression was also observed in STC2 treated *ob/ob* mice (Figure 4B). These data suggest that STC2 may play the role of appetite regulation by modulating hypothalamic neuropeptites expression, especially by inducing anorexic POMC expression.

### STC2 activates STAT3 signaling pathway in the hypothalamus and GT1-7 cells

**Figure 5:**
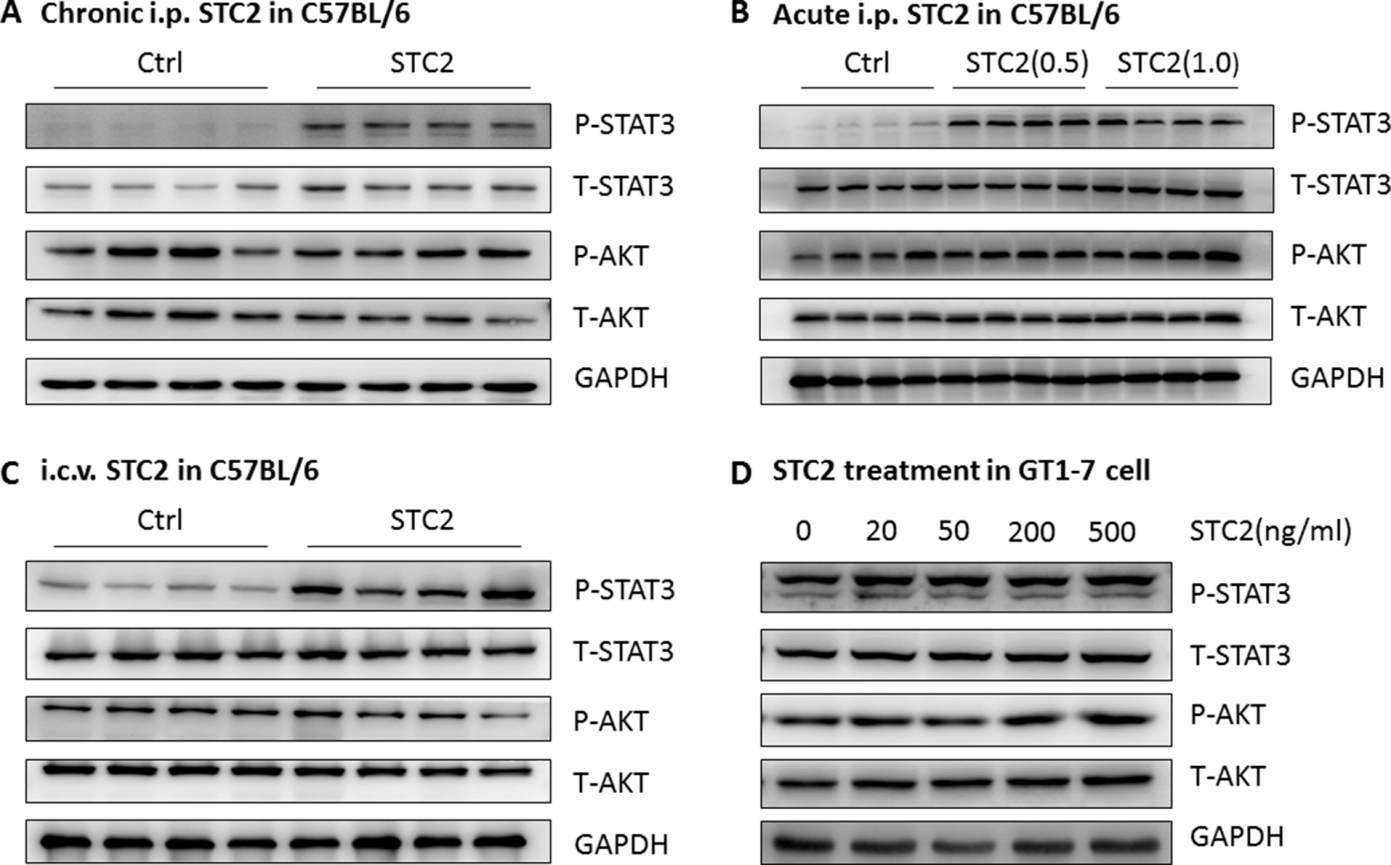
STC2 activates the STAT3 pathway in the hypothalamus and GT1-7 cells (**A**) Western blot analysis demonstrated that chronic systemic administration of STC2 (1.0 mg/kg) to C57BL/6 mice specifically activated the STAT3 pathway in the hypothalamus (*n* = 4). (**B**) Western blots showing the activated hypothalamic STAT3 pathway in C57BL/6 mice receiving a single i.p. injection of STC2 recombinant protein (0.5 mg/kg, 1.0 mg/kg) (*n* = 4). (**C**) Hypothalamic STAT3 signaling pathway in C57BL/6 mice was activated at 30min following an i.c.v administration of STC2 recombinant protein (1.0 mg/kg) (*n* = 4). (**D**) A dramatic activation of STAT3 pathway in hypothalamic GT1-7 cells treated with different dose of STC2 recombinant protein.

Finally, we analyzed potential mechanisms of STC2-induced anorexia effects in mice. Therefore, STAT3 and AKT, two critical molecules involved in food intake [22, 23] were determined. We found that chronic systemic administration of STC2 to C57BL/6 mice significantly activated STAT3 in the hypothalamus, while the AKT signaling pathway was not affected (Figure 5A), indicating that the anorexic effect of STC2 is probably mediated by STAT3 pathway. To examine the acute effect of STC2 on hypothalamic STAT3 pathway, C57BL/6 mice were fasted for 24 hr and received i.p. injection of two dosages of STC2 proteins. The hypothalamus was isolated 30 min after injection. A robust activation of hypothalamic phosphorylated STAT3 was also observed in STC2 treated mice compared with control mice (Figure 5B). We further performed i.c.v. injection of STC2 in C57BL/6 mice to examine STAT3 signaling. Compared with mice treated with PBS, phosphorylated STAT3 was significantly elevated in the hypothalamus in STC2 injected mice at 30 min after injection in a pattern similar to that seen by systemic administration (Figure 5C). Notably, *in vitro* treatment of STC2 also resulted in an activation of STAT3 pathway in GT1-7 cells (Figure 5D). It is well documented that leptin activates intracellular signaling cascades, such as STAT3, through the recruitment of JAK2 to its receptor [24, 25]. Therefore, phosphorylated JAK2 were determined in STC2-treated mice. As a result, we found that neither acute intraperitoneal injection nor intracerebroventricular administration of STC2 could activate JAK2 in hypothalamus of C57BL/6 mice (Supplementary Figure 3A, 3B), suggesting that the down-stream signaling pathways of STC2 might be different from leptin. Collectively, these data suggest that STAT3 pathway may serve as a major signaling pathway in the central nervous system to mediate the anorexia and weight lowering effect of STC2.

## DISCUSSION

In the present study, we identified a novel role of STC2 in the regulation of appetite and body weight. Systemic STC2 administration decreased body weight in C57BL/6 and *ob/ob* mice by inhibiting appetite. STC2 could also inhibit food intake in C57BL/6 mice by i.c.v. injection. Mechanistically, we demonstrated for the first time that STC2 could activate STAT3 signaling pathway in the hypothalamus, as well as in GT1-7 hypothalamic cell line, thus altering the expression of key appetite-regulating neuropeptides in the hypothalamus, including POMC, CART, AgRP, and NPY.

Mammalian STC2 is extensively expressed in various tissues such as liver, pancreas, kidney and skeletal muscle [26, 27].It has been reported that STC2 transgenic mice displayed a dwarf phenotype [28], possibly due to the inhibition of insulin-like growth factor (IGF) axis [29]. On the other hand, STC2 knockout mice were 10–15% larger and grew faster than wild-type littermates from 4 week onward[30], which supported the growth inhibitory effects observed in the transgenic mice. Regretfully, all these studies did not assess food intake of mice, which was also closely relevant to the growth rate and body weight. In this study, we demonstrated that STC2 played a significant role in appetite suppression, thus may contributed to the growth inhibition effect observed in STC2 transgenic mice.

Anorexia is one of the most common symptoms in advanced cancer which affects patients’ response to treatment, as well as survival and quality of life [31, 32]. Cytokines produced either by cancer cells or by immune system appear to play a key role in the pathogenesis of cancer anorexia [16, 17, 32, 33]. These cytokines, by modulating hypothalamic neuronal pathways and disturbing the stringent balance between anorexigenic and orexigenic peptides expression, may lead to suppression of appetite and marked weight loss. The inflammatory cytokines released by immune cells in response to the presence of the cancer, such as interleukin-1β(IL-1β), interleukin-6 (IL-6) and tumor necrosis factor-α (TNF-α), have been shown to play a key role in the disturbances of appetite and body weight control [34–37]. Some cytokines directly derived from cancer cells, such as macrophage inhibitory cytokine-1(MIC-1), could also regulate food intake and energy homeostasis[38]. Interestingly, growing evidence demonstrated that STC2 was induced in most human cancer tissues by hypoxic stress and endoplasmic reticulum(ER) stress in cancer microenvironment [8, 39, 40]. Higher expression level of STC2 was generally correlated with poor prognostic outcome of patients [10–13]. Therefore, we speculate that tumor-derived STC2 might be involved in cancer-induced anorexia, which needs to be determined in the future studies.

Despite significant advances in our understanding of appetite and weight control in cancer patients, effective therapies available are still quite limited. A better appreciation of molecular mechanisms that control appetite and body weight may help in the development of new therapies to improve the survival and quality of life of these patients. Our present study demonstrated that STC2 might be a potential target for improving anorexia and weight loss in cancer patients.

## MATERIALS AND METHODS

### Animal experiments

C57BL/6 and leptin-deficient *ob/ob* mice aged 8–12 weeks were purchased from the Shanghai Laboratory Animal Company (SLAC, Shanghai, China) and Nanjing Biomedical Research Institute of Nanjing University (NBRI, Nanjing, Jiangsu Province, China), respectively. Mice were housed individually for 1 week, then randomly assigned to control or experimental group. STC2 recombinant protein was purchased from Shanghai Boyi Biotechnology Company (Shanghai, China). High-fat diet (45% calories from fat) was purchased from Research Diets (New Brunswick, NJ, USA). For chronic treatment, C57BL/6 mice were injected intraperitoneally (i.p.) with STC2 recombinant protein (1.0 mg/kg) or PBS twice daily (8:00 a.m. and 4:00 p.m.) for 5 consecutive days; ob/ob mice were injected i.p. with STC2 recombinant protein (0.5 mg/kg or 1.0 mg/kg) or PBS twice daily (8:00 a.m. and 4:00 p.m.) for 10 consecutive days. Food consumption and body weight were measured daily at 8:00 a.m. For acute treatment, C57BL/6 mice were fasted for 24hr and then i.p. injected with STC2 recombinant protein (1.0mg/kg) or PBS. For intracerebroventricular (i.c.v.) treatment, cannula placement was performed on C57BL/6 mice 1 week before the injection. Mice were fasted for 24 hr, and then 2 μL (0.5 μg/μl) STC2 recombinant protein or PBS was injected into the lateral ventricle within 10min. All animal experiments were reviewed and approved by the Ethics Committee of Shanghai Jiao Tong University School of Medicine.

### Metabolic studies

C57BL/6 mice were placed in metabolic cages (Columbus Instruments) to assess their O2 consumption, CO2 production and respiratory exchange ratio (RER) within 24 hr (8:00 a.m.–8:00 a.m.).

### Insulin tolerance tests

*ob/ob* mice were injected with regular human insulin at a dose of 0.75 IU/kg body weight after fasting for 6hr. Blood glucose was monitored at the indicated time points via tail bleeding using a portable blood glucose meter (Lifescan, USA).

### Hepatic triglyceride measurement

Liver tissues were homogenized in chloroform/methanol (2:1 v/v) using a tissue grinder. Lipid extracts were prepared by the classical Folch method. Extracts were dried under N2 flow and dissolved in isopropanol. Triglyceride contents were measured using commercial kits (Biovision, USA) according to the manufacturer’s instructions.

### Cell culture

GT1-7 cell line was grown in DMEM (Gibco, USA) supplemented with 10% FBS (Gibco, USA), 100 IU/ml penicillin, and 100 μg/ml streptomycin. Cells were starved in serum-free DMEM overnight and then treated with STC2 recombinant protein or PBS at indicated doses for 30 min.

### RNA isolation and qRT-PCR

Total RNA was extracted from hypothalamic tissues using TRIzol according to the manufacturer’s instructions (Invitrogen, USA). 2 μg of total RNA was reversely transcribed into cDNA using oligo-dT primers (Promega, USA). Quantitative real-time PCR was carried out using SYBR Green Premix Ex Taq (Takara, Japan) on a Light Cycler 480 (Roche, Switzerland). Relative mRNA levels were calculated using the comparative ∆Ct values. The β-actin gene was used as an internal reference for normalization.

### Western blots

Hypothalamic tissues and GT1-7 cells were lysed in radioimmunoprecipitation (RIPA) buffer containing 50 mM Tris-HCl (pH 8), 150 mM NaCl, 5 mM MgCl2, 2 mM EDTA, 1 mM NaF, 1% NP40 and 0.1% SDS. 30μg lysates were loaded onto 10% SDS-PAGE gels and transferred to polyvinylidene difluoride (PVDF) membranes (Millipore, USA) by standard procedures. The antibodies used in western blot include, STAT3 (12640, 9145; Cell Signaling), AKT (13038, 4821; Cell Signaling) and GAPDH (5174; Cell Signaling). The proteins were detected using enhanced chemiluminescence (ECL) reagents (GE LifeScience, UK) according to the manufacturer’s protocal.

### Statistical analysis

All values are expressed as mean ± standard error of mean (SEM). Statistical differences were determined by two-tailed Student’s t test. Statistical significance was shown as *p < 0.05, **p < 0.01 or ***p < 0.001.

## SUPPLEMENTARY MATERIALS FIGURES



## Abbreviations

STC2: stanniocalcin2
DEXA: dual-energy X-ray absorptiometry
BAT: brown adipose tissue
RER: respiratory exchange ratios
CNS: central nervous system
ARC: arcuate nucleus
AgRP: agouti-related protein
NPY: neuropeptite Y
POMC: proopriomelanocortin
CART: cocaine and amphetamine regulated transcript
IGF: insulin-like growth factor
IL-1β: interleukin-1β
IL-6: interleukin-6
TNF-α: tumor necrosis factor-α
MIC-1: macrophage inhibitory cytokine-1
ER: endoplasmic reticulum
i.p.: intraperitoneally
i.c.v.: intracerebroventricular
RIPA: radioimmunoprecipitation
PVDF: polyvinylidene difluoride
ECL: chemiluminescence
SEM: standard error of mean
