# Supplementary material for: Stanniocalcin2 acts as an anorectic factor through activation of STAT3 pathway
Source: Oncotarget. 2017 Jul 20;8(53):91067–75. doi: 10.18632/oncotarget.19412 (PMC5710906; doi:10.18632/oncotarget.19412)
Supplement: Supplementary file 1 [file oncotarget-08-91067-s001.pdf]

Stanniocalcin2 acts as an anorectic factor through activation of STAT3 pathway

SUPPLEMENTARY MATERIALS

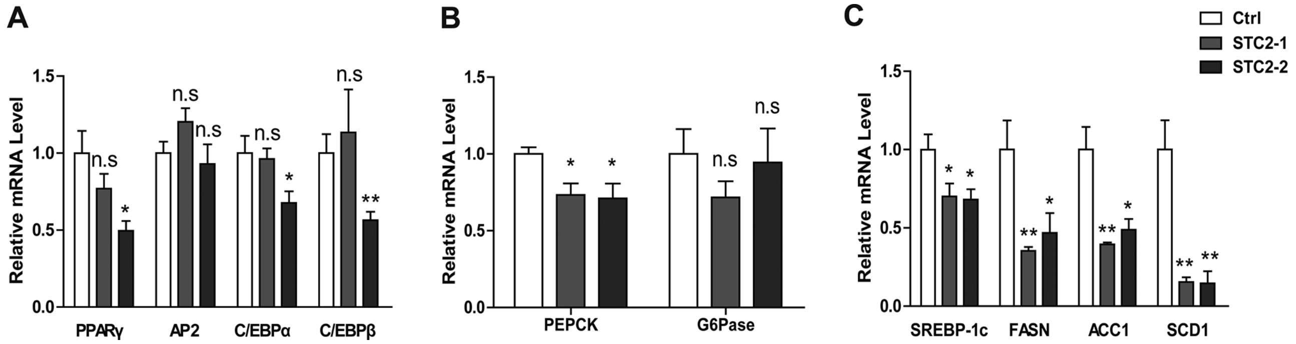

Supplementary Figure 1: STC2 modulates metabolic related genes expression in ob/ob mice. (**A**) Relative mRNA levels of adipogenic genes in epididymal adipose tissue of ob/ob mice that receiving systemic STC2 treatment. (n = 6) (**B–C**) Relative mRNA levels of hepatic gluconeogenic genes (B), triglyceride synthesis related genes (C) in ob/ob mice. (n = 6) Data are expressed as means ± SEM. *P < 0.05; **P < 0.01.

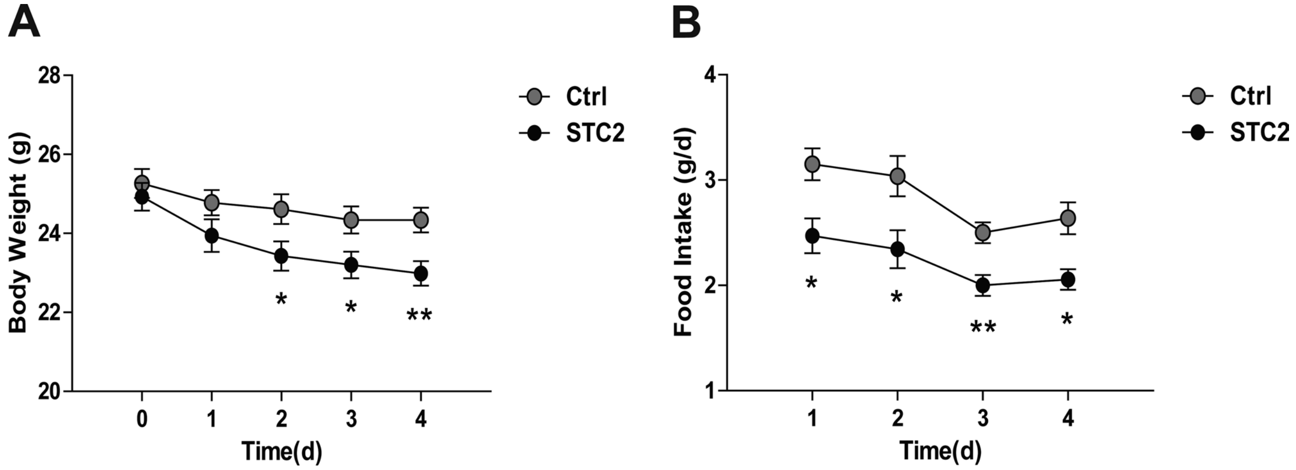

Supplementary Figure 2: STC2 induced anorexia and weight loss in C57BL/6 mice fed with high-fat diet. (**A, B**) Body weight (A) and food consumption (B) of C57BL/6 mice consuming a high-fat diet and administrated intraperitoneally with STC2 recombinant protein (1.0 mg/kg) or PBS. (n = 8) Data are expressed as means ± SEM. *P < 0.05; **P < 0.01.

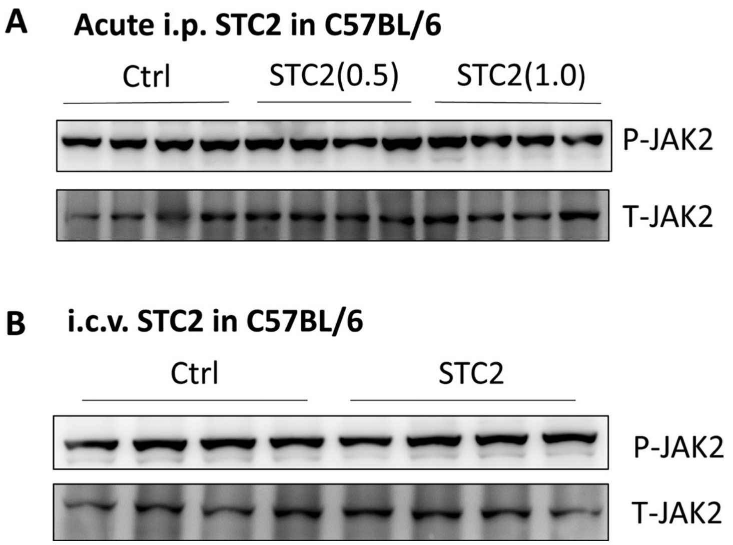

Supplementary Figure 3: STC2 do not affect hypothalamic JAK2 pathway in C57BL/6 and ob/ob mice. (**A**) Western blots of hypothalamic JAK2 pathway in C57BL/6 mice receiving acute intraperitoneal injection of STC2 recombinant protein (0.5 mg/kg, 1.0 mg/kg) (*n* = 4). (**B**) Western blots of JAK2 signaling pathway in the hypothalamus of C57BL/6 mice receiving intracerebroventricular administration of STC2 recombinant protein (1.0 mg/kg) (*n* = 4).
